# Using a vegetation index to assess wetland condition in the Prairie Pothole Region of North America

**DOI:** 10.3389/fenvs.2022.889170

**Published:** 2022-09-07

**Authors:** Brian A. Tangen, Sheel Bansal, Seth Jones, Cami S. Dixon, Amanda M. Nahlik, Edward S. DeKeyser, Christina L. M. Hargiss, David M. Mushet

**Affiliations:** 1 United States Geological Survey, Northern Prairie Wildlife Research Center, Jamestown, ND, United States,; 2 North Dakota State University, School of Natural Resource Sciences, Fargo, ND, United States,; 3 United States Fish and Wildlife Service, Chase Lake National Wildlife Refuge, Woodworth, ND, United States,; 4 United States Environmental Protection Agency, Office of Research and Development, Center for Public Health and Environmental Assessment, Pacific Ecological Systems Division, Corvallis, OR, United States

**Keywords:** ecosystem services, multi-metric index, wetland degradation, wetland restoration, plants

## Abstract

Wetlands deliver a suite of ecosystem services to society. Anthropogenic activities, such as wetland drainage, have resulted in considerable wetland loss and degradation, diminishing the intrinsic value of wetland ecosystems worldwide. Protecting remaining wetlands and restoring degraded wetlands are common management practices to preserve and reclaim wetland benefits to society. Accordingly, methods for monitoring and assessing wetlands are required to evaluate their ecologic condition and outcomes of restoration activities. We used an established methodology for conducting vegetation-based assessments and describe a case study consisting of a wetland condition assessment in the Prairie Pothole Region of the North American Great Plains. We provide an overview of an existing method for selecting wetlands to sample across broad geographic distributions using a spatially balanced statistical design. We also describe site assessment protocols, including vegetation survey methods, and how field data were applied to a vegetation index that categorized wetlands according to ecologic condition. Results of the case study indicated that vegetation communities in nearly 50% of the surveyed wetlands were in *very poor* or *poor* condition, while only about 25% were considered *good* or *very good*. Approximately 70% of wetlands in native grasslands were categorized as *good* or *very good* compared to only 12% of those in reseeded grasslands (formerly cropland). In terms of informing restoration and management activities, results indicated that improved restoration practices could include a greater focus on establishing natural vegetation communities, and both restored and native prairie wetlands would benefit from enhanced management of invasive species.

## 1 Introduction

Wetlands are a globally important natural resource that cover approximately 5–8% of the Earth’s land surface (Millennium Ecosystem Assessment, 2005; Zedler and Kercher, 2005; Mitsch and Gosselink, 2015). The societal value of wetlands is widely recognized and generally linked to the ecological condition or quality of a wetland (Murkin, 1998; Millennium Ecosystem Assessment, 2005; Euliss et al., 2006; Gleason et al., 2011). Despite their intrinsic value to society, wetland loss and degradation are commonly linked with human activities. Wetland loss is most often due to drainage associated with urban development and agricultural practices (McCorvie and Lant, 1993; Johnson et al., 2008; Blann et al., 2009; Yan and Zhang, 2019). To preserve or enhance the delivery of wetland ecosystem services, national policies (e.g., Section 404 of the Clean Water Act, “Swampbuster” provision of 1985 Farm Bill) and efforts to conserve remaining wetlands and restore drained or degraded wetlands have become a focal point for many government agencies (e.g., United States Department of Agriculture Agricultural Conservation Easement Program, United States Fish and Wildlife Service [USFWS] wetland easements) and nongovernmental organizations (e.g., Tori et al., 2002; Gleason et al., 2011). Consequently, techniques are needed to assess these activities and guide future conservation efforts and natural resource management. We provide a brief overview of a variety of wetland assessment techniques and describe a vegetation-based wetland assessment method through presentation of a case study conducted in the Prairie Pothole Region (PPR) of central North America.

### 1.1 Wetland assessments

Effects of anthropogenic activities on aquatic ecosystems, as well as their overall ecologic condition, are typically assessed based on the composition of biotic communities, water quality, hydrologic functions, or degree of anthropogenic impacts (e.g., Karr, 1981; Brinson, 1993; Kerans and Karr, 1994; DeKeyser et al., 2003; Post van der Burg and Tangen, 2015; McMurry et al., 2016; Schwarz et al., 2018). Approaches used to assess various pollutants of aquatic systems (e.g., streams, rivers, wetlands) often focus on chemical analyses (e.g., nutrients, metals, agrichemicals) or various water-quality parameters (e.g., dissolved oxygen, pH, turbidity). Water-quality sampling can be useful for identifying elevated or harmful levels of metals, nutrients, or agrichemicals by comparing observed levels to standards that typically are established by state regulatory agencies. While water-quality assessments can be informative, they do have limitations because standards developed for lakes or streams often do not apply to wetlands; many wetlands have short periods of inundation and even large wetlands dry completely during extended drought (Euliss et al., 2004; Kentula et al., 2020). Concentration of some water-quality parameters can vary widely, both within and among years, because of concentration and dilution associated with precipitation, runoff, and evapotranspiration (Euliss et al., 2014; Mushet et al., 2015; Hayashi et al., 2016). Connection to groundwater (e.g., recharge, discharge), which varies greatly among wetlands, also can have considerable effects on water chemistry (Goldhaber et al., 2011; LaBaugh et al., 2018; Levy et al., 2018). Moreover, many potential contaminants, such as agrichemicals, can have short life spans or residence times, and in the case of riverine systems, contaminants are transported downstream from the source.

Rather than concentrating on specific contaminants or water-quality parameters, many assessments focus on the overall ecologic condition, or “health,” of aquatic systems. This approach often involves the development and application of multi-metric indices (e.g., Index of Biotic Integrity; Karr, 1981), in which biotic communities such as fish, macroinvertebrates, or plants are surveyed across an observed stressor gradient, and biological response variables or metrics are identified (e.g., Burton et al., 1999; Lopez and Fennessy, 2002; DeKeyser et al., 2003; Seilheimer and Chow-Fraser, 2006; Lu et al., 2019). Effective metrics that display a predictable response to stressors are then used to reflect the condition of a site by calculating an overall index score, which can then be used to define condition categories such as very poor, poor, fair, good, or excellent. Index scores associated with each condition category are typically determined based on community attributes observed at minimally impacted reference sites. In addition to standard, single-community indices, recent research has explored the utility of multi-community (e.g., plants + birds, plants + phytoplankton + invertebrates) indices (e.g., Gronke, 2004; Wilson and Bayley, 2012), as well as a modeling framework for developing biomonitoring tools (Bolding et al., 2020). These methods, however, may require additional financial resources, as well as more time, labor, and expertise (Wilson and Bayley, 2012; Bolding et al., 2020). Conversely, less complicated indices that require fewer resources and rely on relatively few metrics (e.g., floristic quality) have proven effective for assessing wetland condition, especially when time and resources are limited (Guntenspergen et al., 2002; Lopez and Fennessy, 2002; Mack, 2006; Rebelo et al., 2009; Bourdaghs, 2012; Hargiss et al., 2017). Regardless of the approach chosen, assessing the condition of wetlands can be challenging due to the highly variable nature of many systems (Wilcox et al., 2002; Tangen et al., 2003; Langer et al., 2018; Mushet et al., 2018; McLean et al., 2020; Mushet et al., 2020). Therefore, results of wetland assessments must be viewed in light of current and past climate (e.g., drought) and hydrologic conditions (depth, permanence), which directly affect wetland characteristics (e.g., vegetation, chemistry) regardless of anthropogenic disturbances (Kantrud and Newton, 1996; Wilcox et al., 2002; Euliss et al., 2004; Euliss and Mushet, 2011).

While studies that describe the development and validation of various approaches and metrics for assessing aquatic systems are common, there are far fewer studies that describe the practical application of existing biotic indices with the goal of informing management or conservation practices. We present a regional case study that uses established methods to assess plant communities of wetlands embedded within grasslands managed for wildlife habitat, species conservation, and outdoor recreation.

### 1.2 Prairie Pothole Region wetlands: A case study

The PPR of North America is widely recognized for its agricultural production, abundant wetland resources, and concentration of lands enrolled in conservation programs. Additionally, tracts of native prairie that have not been tilled still exist in the western PPR (e.g., Missouri Coteau ecoregion), although these lands typically are subjected to grazing and frequently impacted by invasive plants. Thus, the region is well suited for developing and demonstrating methods for assessing the ecologic condition of restored, degraded, and natural (although impacted) wetlands. We describe a proven methodology for assessing wetland ecological condition (DeKeyser et al., 2003; Hargiss et al., 2008; Hargiss, 2009; Hargiss et al., 2017) and demonstrate the utility of the method through description of a recent (c. 2020–2021) case study from the PPR (Tangen et al., 2019; Jones, 2021). While the sample methods, plant-based index, and case study are specific to wetlands of the PPR, the general methodology or approach could be adapted to other inland wetlands with varying degrees of effort (e.g., modified sample design, identifying alternative metrics or scoring criteria).

#### 1.2.1 Prairie Pothole Region

**FIGURE 1:**
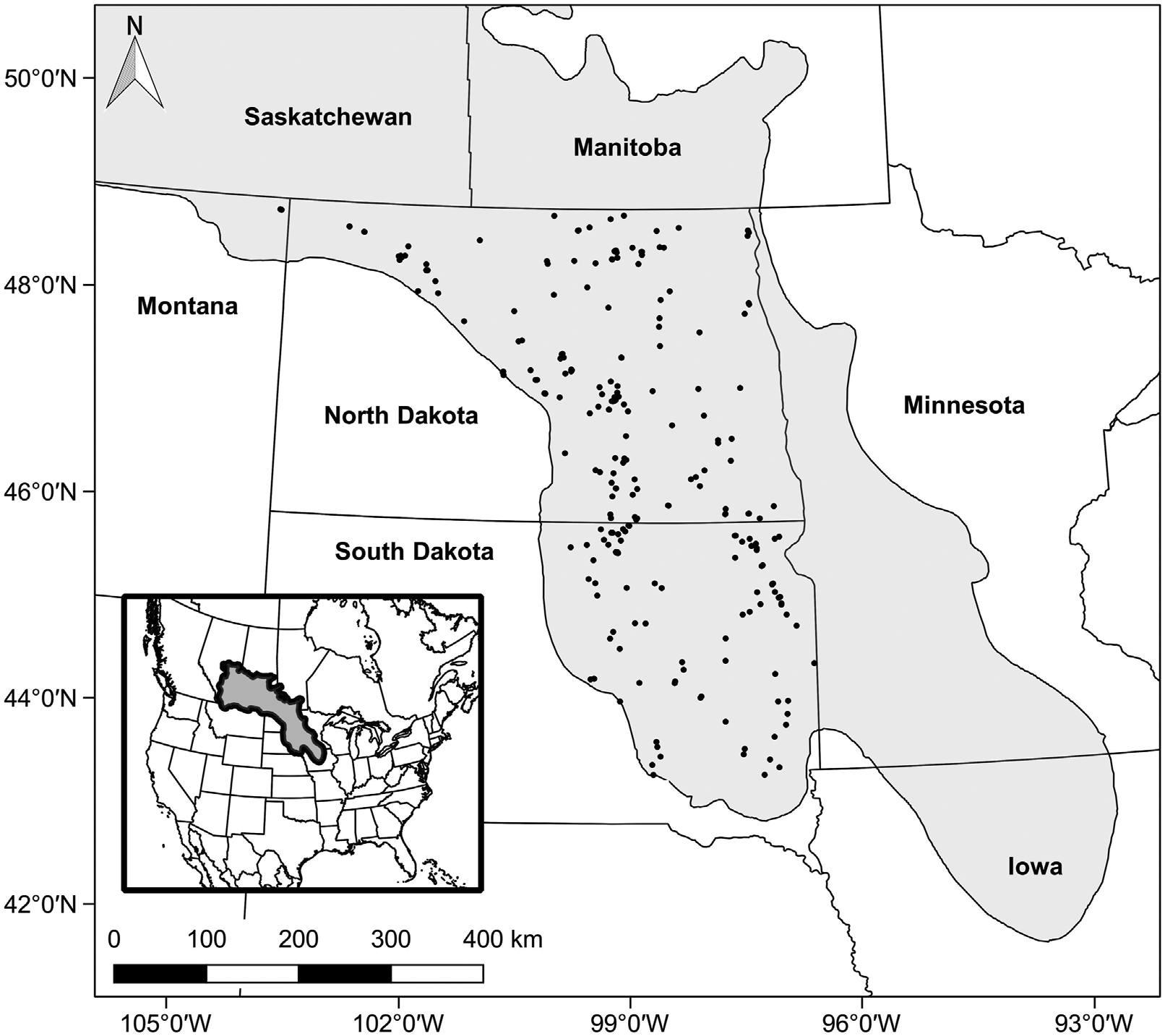
The Prairie Pothole Region of North America (shaded area). Dots indicate the location of wetlands selected for the 2020–2021 case study in the United States portion of the region.

The PPR (Figure 1) covers nearly 800,000 km^2^ of central North America, including portions of five United States states and three Canadian provinces (Euliss et al., 2006; Dahl, 2014). The PPR is distinguished by millions of small, depressional, mineral-soil wetlands, hereafter referred to simply as wetlands. Wetlands in the PPR provide a range of ecosystem services that include wildlife habitat, carbon sequestration, flood mitigation, filtration of pollutants, groundwater recharge, nutrient retention, and recreational opportunities (Winter and Rosenberry, 1995; Knutsen and Euliss, 2002; Euliss et al., 2006; Gleason et al., 2008; Badiou et al., 2011; Gleason et al., 2011). Wetlands of the region are particularly well-known for providing breeding, brood-rearing, and migration stop-over habitats for a large proportion of North America’s migratory water birds (Batt et al., 1989; Niemuth et al., 2006; Skagen et al., 2008; Niemuth et al., 2018). Estimates suggest that greater than half of the wetlands in the United States have been lost due to human activities, and a considerable number of those that remain are degraded as a result of land-use and climate change (DeKeyser et al., 2003; Johnston, 2013; Dahl, 2014; U.S. Environmental Protection Agency, 2016a; Bourdaghs et al., 2019). The intrinsic value of wetlands to society has led to the PPR becoming a focal region for conservation programs in the United States and Canada. However, despite the considerable resources committed to restoring and preserving wetlands in the PPR, relatively little effort has been dedicated to studying the outcomes of these activities (Galatowitsch and Bohnen, 2020, 2021). Broad studies conducted at state and National levels, which included PPR wetlands, have assessed wetland quality for specific regions and wetland types, but these studies typically lack the resolution required to characterize specific restoration or management practices, as well as particular wetland subclasses (U.S. Environmental Protection Agency, 2016a; Bourdaghs et al., 2019). Thus, future conservation activities, as well as ongoing management, would benefit from recurring assessments of the overall ecologic condition of both natural and restored wetlands in the PPR.

#### 1.2.2 Wetland conservation in the Prairie Pothole Region

Thousands of square kilometers of land in the PPR have been protected from development through land acquisition, establishment of conservation easements, or restoration from a cropland setting to a grassland setting through various governmental and non-governmental conservation programs (Gleason et al., 2011; Doherty et al., 2013; Walker et al., 2013; Niemuth et al., 2014; Dixon et al., 2019). Often, wetland restoration occurs on these protected lands, typically consisting of 1) disrupting surface or subsurface drainage systems to reestablish natural hydrology, and 2) reseeding surrounding uplands (i.e., croplands) to grassland species (Gleason et al., 2011; Dixon et al., 2019). In many instances, intact (i.e., not drained) wetlands embedded within croplands are not specifically targeted for restoration but are essentially restored when adjacent croplands are converted to grasslands. Restoration of both drained and intact wetlands, however, generally does not involve seeding or planting wetland vegetation, or removing accumulated sediment; rather, wetland species typically are established through natural recolonization from remnant seed banks or wind-blown seeds (Galatowitsch and van der Valk, 1996a; b; Mulhouse and Galatowitsch, 2003; Gleason et al., 2011; Smith et al., 2016). This lack of targeted restoration and management can result in reduced water depths from sediment accretion and low-diversity, wetland plant communities composed of annual or invasive species (Kantrud and Newton, 1996; Gleason and Euliss, 1998; Mulhouse and Galatowitsch, 2003; Aronson and Galatowitsch, 2008; Smith et al., 2016; Galatowitsch and Bohnen, 2021).

Although formal assessments of specific restoration activities are sparse, plant communities of restored wetlands in the PPR have been shown to differ from those of native-prairie wetlands that have not been directly affected by tillage and cropping (e.g., Galatowitsch and van der Valk, 1996b; Seabloom and van der Valk, 2003; Aronson and Galatowitsch, 2008; Laubhan and Gleason, 2008; Paradeis et al., 2010; Smith et al., 2016). Restored wetlands in the PPR are often colonized by fewer species than are found in natural sites, their wet-prairie and sedge-meadow zones generally do not redevelop, and many become dominated by invasive perennial species such as *Phalaris arundinacea* (reed canarygrass) and *Typha × glauca* (hybrid cattail) (Seabloom and van der Valk, 2003; Aronson and Galatowitsch, 2008). Based on generalizations from the previous studies in the PPR, periodic regional assessments that target sites of interest could benefit conservation efforts by providing information related to specific wetland types, management practices, or regions.

Within the PPR, the USFWS National Wildlife Refuge System (NWRS) manages thousands of square kilometers consisting of lands such as National Wildlife Refuges (NWRs) and Waterfowl Production Areas (WPAs) (Dixon et al., 2019). These conservation lands preserve and restore grassland/wetland complexes to support the conservation of wildlife, and provide habitat for migratory birds such as waterfowl. In recent years, a focus of the USFWS has been on restoring and reconstructing the grassland portion of these complexes (e.g., Gannon et al., 2013; Igl et al., 2018; Dixon et al., 2019), with less emphasis on the embedded pothole wetlands. At a national level, although a vegetation-based condition assessment indicated that 80% of the wetland area in the United States Interior Plains, which partially overlays the PPR, was in good or fair condition, 19% was considered poor (U.S. Environmental Protection Agency, 2016a). Conversely, a regional vegetation assessment indicated that 82% of the wetlands in western Minnesota were in poor or fair condition, while only 18% were considered good or exceptional (Bourdaghs et al., 2019). These results highlight the need to assess the outcomes of current and previous management practices, in terms of the ecologic condition of wetlands, to inform management strategies for improving the condition and functioning of restored and natural wetlands.

#### 1.2.3 Biotic indices in the Prairie Pothole Region

All manner of biotic communities of wetlands, along with geochemistry and soils, have been widely studied, but plant and invertebrate communities generally have been promoted as potential indicators of wetland ecologic condition (DeKeyser et al., 2003; Tangen et al., 2003; Hargiss et al., 2008; Gleason and Rooney, 2017; Hargiss et al., 2017; Preston et al., 2018). Aquatic invertebrates are an important component of the food chain, and various taxa have been shown to be sensitive to disturbance or pollution in aquatic systems such as streams (e.g., Kerans and Karr, 1994; Fore et al., 1996; Morley and Karr, 2002). Plants provide habitat for a wide variety of birds, invertebrates, and other wildlife, with species composition closely coupled with soils, hydrology, and water chemistry. Plant communities often respond in predictable patterns to anthropogenic impacts; thus, they also are well-suited to function as indicators of wetland condition. Aquatic invertebrates of wetlands have shown inconsistent and variable responses to stressors, and efforts to incorporate them into biotitic indices have proven largely ineffective (Tangen et al., 2003; Hentges and Stewart, 2010; Batzer, 2013; Gleason and Rooney, 2017; Preston et al., 2018).

Conversely, vegetation-based assessments have resulted in the development and implementation of biotic indices for assessing wetland condition (Kantrud and Newton, 1996; DeKeyser et al., 2003; Reiss, 2006; Hargiss et al., 2008; Reiss et al., 2010; Rooney and Bayley, 2012; Wilson and Bayley, 2012; Wilson et al., 2013). DeKeyser et al. (2003) developed the index of plant community integrity (IPCI) for assessing the quality of wetland plant communities, which can be a surrogate for overall wetland condition. Hargiss et al. (2008) demonstrated the validity of the IPCI as a tool for assessing various classes of wetlands throughout the PPR, indicating its utility for informing wetland management and conservation practices. We synopsize established methods and procedures developed in North and South Dakota for using plants to assess inland wetlands of the PPR. The utility of these vegetation-based assessments is demonstrated through presentation of a case study performed in the PPR of North America, although the information provided should be applicable elsewhere with appropriate modifications. The overall goal was to inform and guide wetland restoration and management, while the overarching objective of this paper was to provide a real-world example of the application of existing methodologies, while discussing important aspects to consider when utilizing and adapting biotic indices to a diversity of aquatic systems.

## 2 Methods

### 2.1 Selection of wetlands

The PPR case study focused on temporarily and seasonally ponded wetlands (classification of Stewart and Kantrud, 1971) located on native prairie or reseeded (i.e., restored) former croplands managed by the USFWS (i.e., NWRs and WPAs). Wetlands were classified and identified based on wetland polygons from a modified USFWS National Wetlands Inventory geodatabase (see Tangen et al., 2019). Selection of study wetlands was constrained to the North Dakota, South Dakota, and Montana portions of the United States PPR (Figure 1). These relatively small (<1.0 ha) and shallow (<1 m) wetlands generally have two to three vegetation zones and make up roughly 90% of wetlands throughout the PPR (Stewart and Kantrud, 1971; Niemuth et al., 2010; Dahl, 2014). Spatially balanced designs for populations that are unevenly distributed across the landscape provide spatially distributed samples that are more likely to be representative of the population than the commonly used random sampling approach (Dunn and Harrison, 1993; Stevens and Olsen, 2004; Olsen et al., 2012). Thus, we selected study wetlands using a generalized random tessellation stratified (GRTS) sampling design, following the approach of the United States Environmental Protection Agency’s National Wetland Condition Assessment (Stevens and Olsen, 2004; Stevens and Jensen, 2007; U.S. Environmental Protection Agency, 2016b; Olsen et al., 2019). This approach generated a randomly selected, but spatially balanced distribution of 250 wetlands stratified by hydrologic regime (i.e., temporarily, seasonally ponded) and sample year (i.e., Year 1 [2020] and Year 2 [2021]; Tangen et al., 2019). Equal number of wetlands were targeted for each class and sample year. Two hundred of the selected wetlands were designated as primary sample sites, while the remaining 50 were designated as alternates for use when the primary sites were deemed not appropriate for sampling (e.g., misclassified, inaccessible). Of the 200 sampled wetlands, 48 (13 temporarily ponded, 35 seasonally ponded) and 152 (46 temporarily ponded, 106 seasonally ponded) were located within native prairie and reseeded croplands, respectively. Site selection was performed using the ‘spsurvey’ package (Kincaid and Olsen, 2019) in R (R version 3.0.1; R Core Development Team, Vienna).

### 2.2 Plant surveys

**FIGURE 2:**
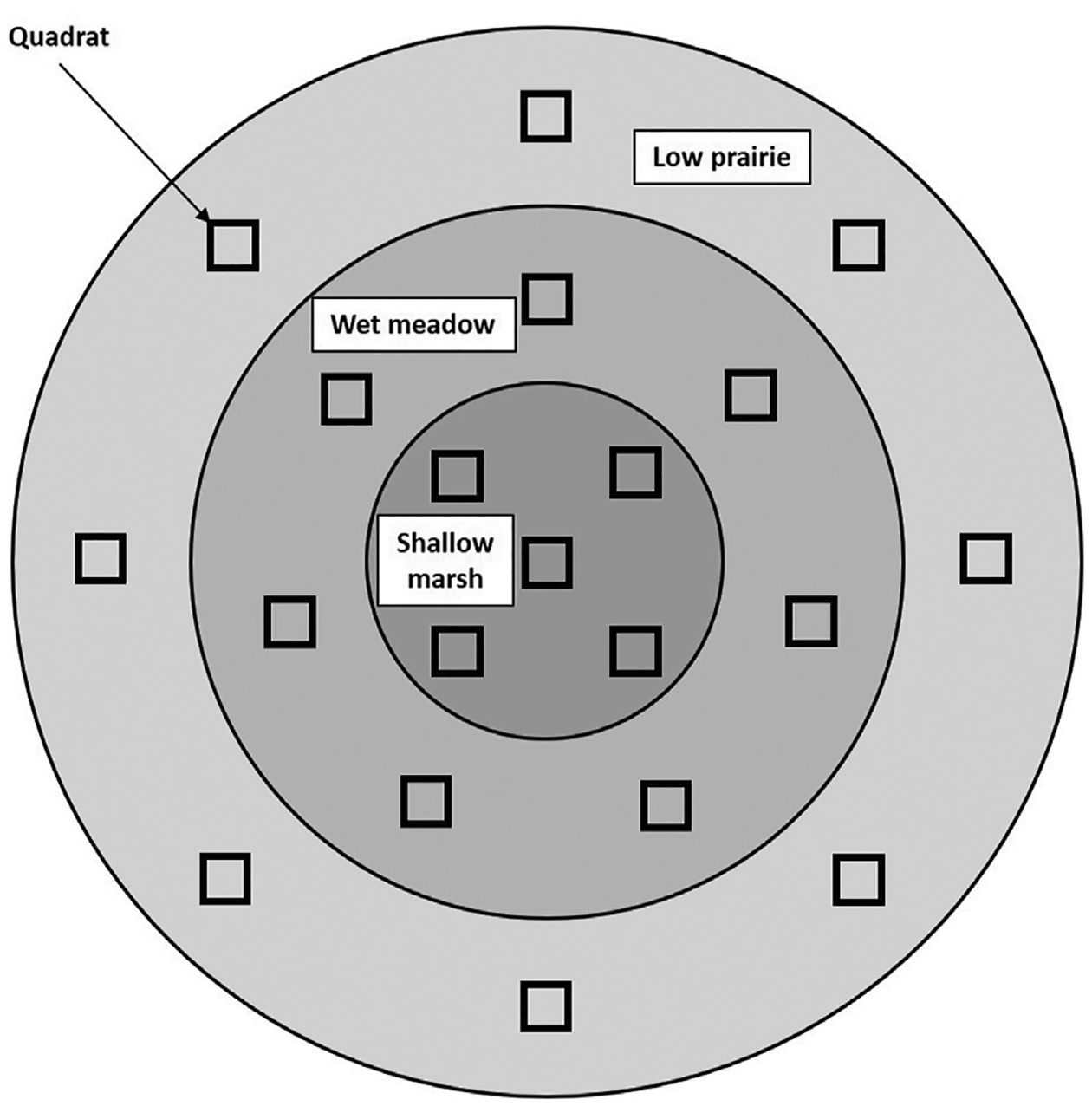
General vegetation sample layout for quadrats in the low-prairie, wet-meadow, and shallow-marsh zones of seasonally ponded wetlands of the Prairie Pothole Region of North America (see DeKeyser et al., 2003; Hargiss, 2009). Temporarily ponded wetlands have a similar layout, but only have an exterior low-prairie zone and a central wet-meadow zone.

Plant surveys were performed during summer when most plants were mature enough to be identified to species. One hundred wetlands were surveyed during each of the 2 years of the study (2020–2021; Jones, 2021). Survey and inventory procedures followed an established quadrat method for PPR wetlands (DeKeyser et al., 2003; Hargiss et al., 2008). Upon arrival at a site, the primary concentric wetland vegetation zones (Stewart and Kantrud, 1971) were delineated. Both temporarily and seasonally ponded wetlands had an exterior low-prairie zone and an interior wet-meadow zone (central zone for temporarily ponded); additionally, seasonally ponded wetlands had a central, shallow-marsh zone. For seasonally ponded wetlands, eight quadrats (1 m^2^) were evenly distributed throughout the low-prairie zone; seven in the wet-meadow zone and five in the shallow-marsh zone. For temporarily ponded wetlands, eight quadrats were distributed throughout the low-prairie zone, and seven quadrats were distributed in the wet-meadow zone. Quadrats were centered in the interior and exterior vegetation zones, and oriented in a spiraled configuration in the central vegetation zone (DeKeyser et al., 2003; Hargiss, 2009; Figure 2). When open water was present in the central zone, quadrats were distributed proportionally to the area of open water and emergent vegetation following DeKeyser et al. (2003) and Hargiss (2009). The entire wetland was surveyed regardless of area; thus, spacing between quadrats varied among wetlands. Plant species within each quadrat were identified. In addition to the primary species within the sample quadrats, species located between, but not within, quadrats were recorded and used to determine IPCI scores (Hargiss et al., 2008). Additional information describing quadrats also was documented, including the percentage of the quadrat covered by standing dead vegetation, open water, or bare ground, and litter thickness and water depth.

### 2.3 Wetland condition

Data from the plant surveys were used to assess wetland condition using metrics developed for the prairie wetland IPCI by DeKeyser et al. (2003), along with modified metric value ranges and scoring criteria presented by Hargiss et al. (2008). There are several established approaches to metric scoring in the literature that include using ordinal classes (e.g., DeKeyser et al., 2003; Hargiss et al., 2008) and continuous scoring (e.g., U.S. Environmental Protection Agency, 2016b; Magee et al., 2019). We chose to use the established ordinal-class approach to metric scoring for the PPR from DeKeyser et al. (2003) and Hargiss et al. (2008) instead of developing new indices and scoring criteria that a continuous scoring approach would necessitate. In total, nine plant community metrics were used to assign a condition score to each temporarily and seasonally ponded pothole wetland, with a maximum possible score of 99. The nine metrics represent various aspects of species richness, species composition, disturbance tolerance, and floristic quality (Table 1). The species richness and composition metrics focus on the number of native perennial species or genera, and the proportion of annual, biennial, and introduced species. Stress tolerance of native species was determined using a coefficient of conservatism, or ‘C-value,’ following The Northern Great Plains Floristic Quality Assessment Panel (2001). The C-value was used to calculate three metrics based on a plant’s stress tolerance, and one metric based on a floristic quality index (DeKeyser et al., 2003), which was calculated as the average C-value multiplied by the square root of the total number of native plant species (Table 1). Metric scores for each wetland were summed, and wetlands were categorized as very poor, poor, fair, good, or very good following Hargiss et al. (2008; Table 2).

### 2.4 Data analysis

Condition classes were assigned to each of the 200 sampled wetlands, and the unweighted results were summarized by number and percent of wetlands in each condition category. Condition classes were determined using all species identified, including those not located within the sample quadrats. Because these results are based on the number of wetlands in each category, there are no associated confidence intervals.

## 3 Results

The wetland selection process resulted in an equal number of temporarily and seasonally ponded wetlands. Wetland classification based on hydrologic and vegetation conditions, however, is temporally variable based on weather and climate (Euliss et al., 2004); thus, spatial wetland databases are inherently associated with some classification variability and error. Based on onsite classification by field personnel (following Stewart and Kantrud, 1971), 59 pothole wetlands were treated as temporarily ponded (two vegetation zones) and 141 as seasonally ponded (three vegetation zones) based on vegetation conditions during 2020–2021. Moreover, 24 of the initial 200 wetlands were replaced with alternates due to factors such as misclassification and difficulty of access. Thus, it is important to select alternate wetlands during the site selection process to avoid potential bias when replacing sites deemed not appropriate for sampling.

**FIGURE 3:**
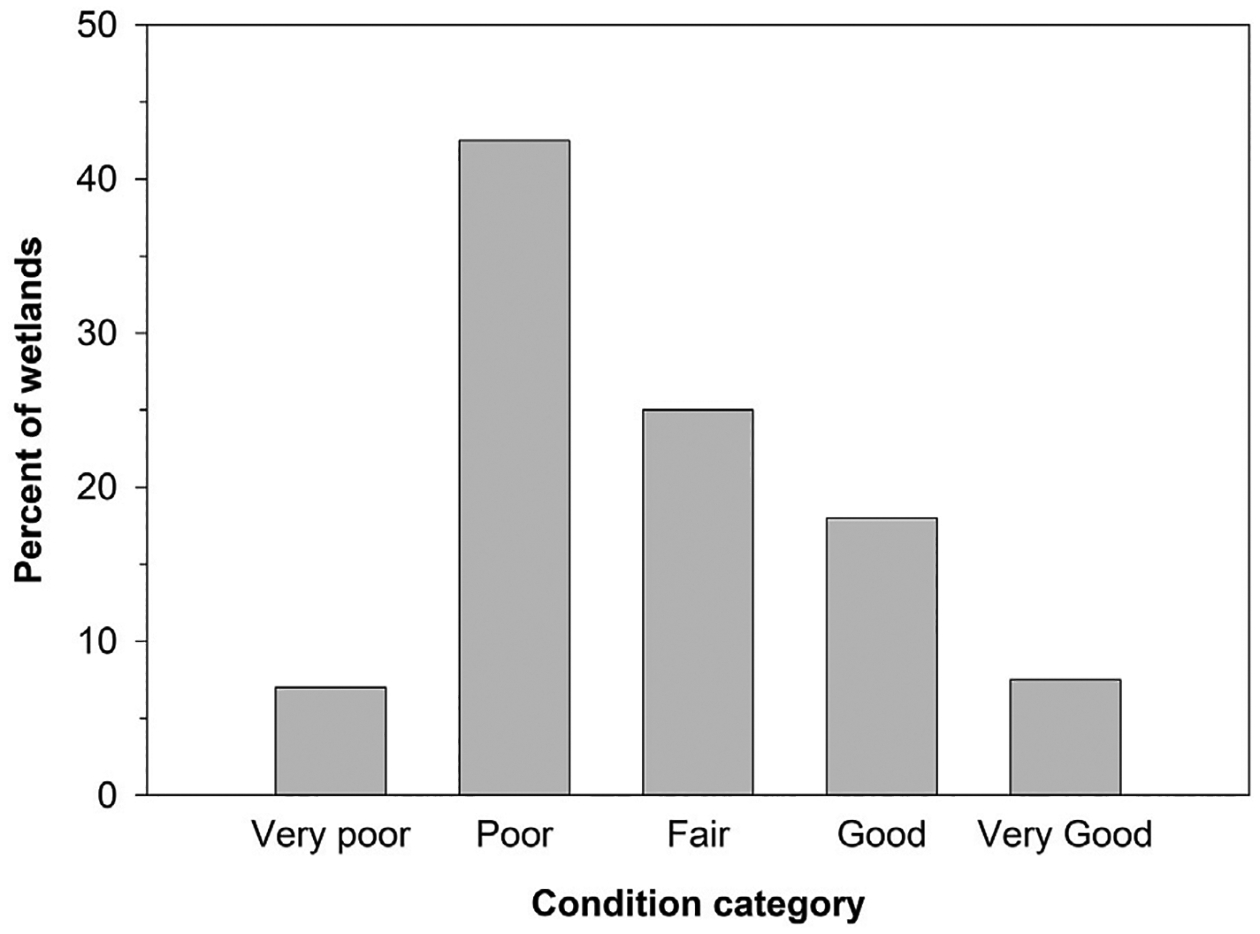
Percent of sampled (2020–2021) wetlands assigned to each Index of Plant Community Integrity condition category. Data represent 59 temporarily ponded and 141 seasonally ponded wetlands located within native prairie and reseeded grasslands in the United States portion of the Prairie Pothole Region of North America.

**FIGURE 4:**
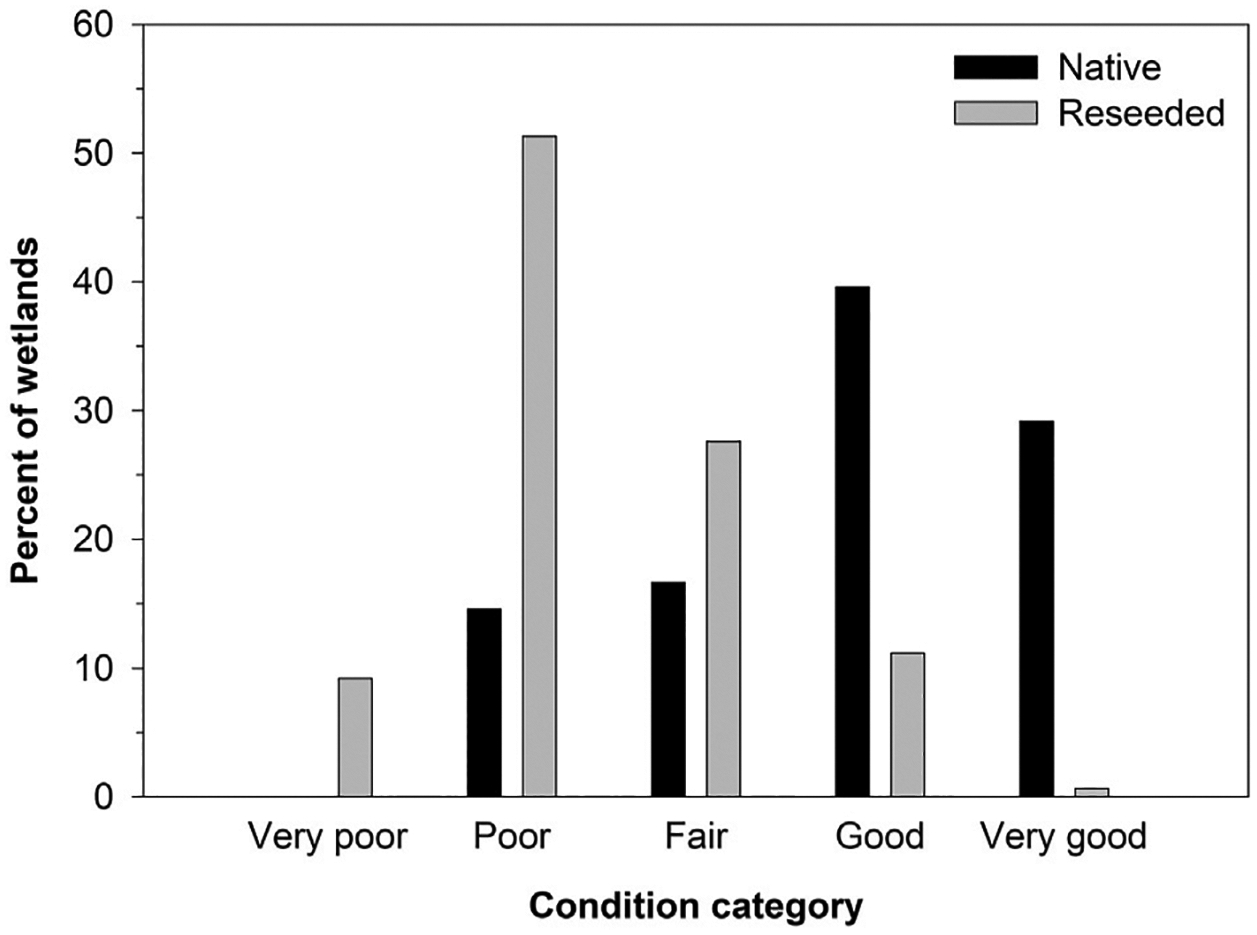
Percent of sampled (2020–2021) wetlands within 48 native prairie and 152 reseeded grasslands assigned to each Index of Plant Community Integrity condition category. Data represent 59 temporarily ponded and 141 seasonally ponded potholes in the United States portion of the Prairie Pothole Region of North America.

When the 200 wetlands from both classes and years were combined, IPCI scores indicated that 49.5% were in very poor or poor condition, 25.0% were in fair condition, and 25.5% were in good or very good condition (Figure 3). When segregated by grassland type, the average (± standard deviation) IPCI score of the wetlands in native grasslands (64 ± 24) was nearly twice that of wetlands in reseeded grasslands (34 ± 21). Approximately 70% of wetlands in native grasslands were categorized as good or very good, while only 12% of pothole wetlands in reseeded grasslands were categorized as good or very good (Figure 4). Conversely, 15% of wetlands in native grasslands were categorized as poor, while >60% of wetlands in reseeded grasslands were categorized as poor or very poor. No wetlands in native grasslands were categorized as very poor (Figure 4). When segregated by wetland class, 54% of seasonal and 42% of temporarily ponded wetlands were categorized as fair, good, or very good. Correspondingly, 46% of seasonally ponded and 58% of temporarily ponded wetlands were categorized as poor or very poor. Common (i.e., present in ≥50% of wetlands) plants identified in study wetlands included invasive or non-native species such as Canada thistle (*Cirsium arvense*), smooth brome (*Bromus inermis*), Kentucky bluegrass (*Poa pratensis*), reed canarygrass, and hybrid cattail (Jones, 2021). Moreover, disturbed wetlands with low condition scores typically were dominated by smooth brome, Kentucky bluegrass, reed canarygrass, and hybrid cattail (Jones, 2021).

## 4 Discussion

Through presentation of a case study (see Tangen et al., 2019; Jones, 2021), we demonstrated a method for assessing wetland condition using an established biotic index based on vegetation (DeKeyser et al., 2003; Hargiss et al., 2008). In doing so, we also used a proven method for selecting a representative, spatially balanced sample of wetlands across a broad spatial area (Stevens and Olsen, 2004; Stevens and Jensen, 2007; U.S. Environmental Protection Agency, 2016b; Olsen et al., 2019). While the case study was specific to the PPR, the overall methodologies and approach are applicable to other areas and wetland types, although modifications to metrics and scoring criteria likely would be required.

### 4.1 Informing wetland management

Wetland condition assessments can provide relevant and timely data to wetland managers and conservation organizations that can be used to inform policy development, guide the allocation of resources, frame the problem of invasive and non-native plants, determine if management and restoration objectives are met, and support overall conservation efforts such as land procurement and habitat improvement. Using data from this field study, Jones (2021) identified USFWS wetland management districts located in north-west North Dakota and north-east South Dakota as having the highest quality (i.e., greatest average IPCI/species richness scores) wetland vegetation communities. In general, these high-quality areas were characterized by greater amounts of native grasslands and less agricultural soil disturbance than areas with low-quality communities. Conservation organizations can use such information to inform and guide management and conservation efforts. For example, areas characterized by relatively high-quality wetlands could be prioritized for establishment of conservation easements to protect wetlands from anthropogenic activities. Similarly, areas characterized by relatively low-quality wetlands could be prioritized for restoration or invasive species management. Information gained from these types of assessments also can be used to refine habitat management practices or prioritize management of specific areas or wetland types associated with degraded habitat conditions.

Results of the PPR case study suggested that plant communities from approximately one-half of the wetlands included in this study were in very poor or poor condition, while only 25% were in good or very good condition. Jones (2021) showed that the wetlands from the PPR case study with low index scores (e.g., very poor, poor) were characterized by fewer plant species than those with larger index scores (e.g., good, very good). Moreover, wetlands with low index scores tended to be dominated by invasive or non-native species.When results were examined separately for wetlands within native and reseeded grasslands, however, it was evident that most of the restored wetlands were in poor condition, while most of the natural wetlands were in relatively good condition (Figure 4). Nevertheless, roughly 30% of the wetlands embedded within native grasslands were characterized by vegetation communities considered poor or fair, suggesting that both restored and native prairie wetlands would benefit from enhanced management of invasive species. Moreover, results suggest that the USFWS could benefit from allocation of additional resources for monitoring managed lands and assessing restoration and management practices.

These general results, along with findings of previous studies (Kantrud and Newton, 1996; Mulhouse and Galatowitsch, 2003; Aronson and Galatowitsch, 2008; Smith et al., 2016; Galatowitsch and Bohnen, 2021), indicate that wetland restoration techniques and management could benefit from practices that focus not only on re-establishing natural wetland plant communities, but on controlling invasive species (e.g., prescribed burns, herbicide treatments, or grazing). For instance, restoration practices in the PPR, which often focus on re-establishing hydrology and restoring croplands to grassland, could be modified to include seeding native wetland plants when possible. Currently, wetland plants typically establish through natural colonization. While not a common practice, Smith et al. (2016) suggested that removal of accumulated sediment may result in greater quality plant communities for restored wetlands; thus, restoration efforts also could incorporate this practice as well.

### 4.2 Application to other areas and wetland types

Biotic indices based on a variety of organisms (e.g., plants, invertebrates, fish) have been developed for numerous aquatic systems (e.g., Karr, 1981; Lopez and Fennessy, 2002; Seilheimer and Chow-Fraser, 2006; Lu et al., 2019; Magee et al., 2019). The plant-based IPCI used for this case study has proven useful for assessing wetlands of the PPR (DeKeyser et al., 2003; Hargiss et al., 2008). These concepts and methods should be easily adapted elsewhere. Specific metrics and scoring criteria used for the IPCI (Tables 1, 2), however, likely would not be appropriate for wetland systems outside of the PPR, or even other wetland classes within the PPR (e.g., permanent pond, fen). Furthermore, our metrics rely on presence/absence of species and do not incorporate abundance data, the incorporation of which may result in a more powerful multi-metric index. Thus, initial efforts to adapt the IPCI (or other indices) to other areas and wetland types would require assessing the utility of the PPR metrics, and possibly the development of alternative metrics and scoring criteria. This process may include the sampling of wetlands across a broad degradation gradient to validate existing metrics, or to identify new metrics, including abundance metrics, that display a predictable response to disturbance. Reference wetlands (i.e., least disturbed, best available; Herlihy et al., 2019) also may have to be sampled to facilitate development of index scoring ranges for the various condition categories (e.g., poor, fair, good).

To maximize the provisioning of ecosystem services, conservation personnel require methodologies to assess wetland condition with the purpose of supporting wetland conservation and management. We presented a case study from the PPR to demonstrate the utility of using an existing plant-based biotic index applied with a probabilistic sampling design to assess wetland condition over a broad geographic setting and over different management settings. Methods used for the case study, including site selection, field sampling protocols, plant metrics, and scoring criteria, were developed specifically for PPR wetlands; however, they can provide a baseline for subsequent studies and can be adapted for a variety of wetlands. We also used results of the case study to demonstrate how wetland assessments can inform management and discussed considerations for adapting the method for other systems. Results of this and other studies suggest that plant community characteristics can be appropriate surrogates for assessing the ecologic condition of wetlands, but natural variability and unique traits of specific wetland types should be considered.

## Figures and Tables

**TABLE 1:** Index of Plant Community Integrity metric value ranges for scores of 0, 4, 7, and 11 for temporarily ponded and seasonally ponded wetlands of the Prairie Pothole Region of North America (see Hargiss et al., 2008).

Metric	0	4	7	11
Temporarily ponded				
Species richness of native perennials	0–16	17–23	24–40	≥41
Number of Genera of native perennials	0–11	12–19	20–26	≥27
Number of native grass and grass-like species	0–8	9–10	11 −15	≥16
Percentage of annual, biennial, and introduced species	≥41.1	35.1 −41.0	27.1–35.0	0.0–27.0
Number of native perennial species in wet-meadow zone	0–7	8–10	11 −13	≥14
Number of species with C value ≥ 5^a^	0–4	5–11	12–16	≥17
Number of species in the wet-meadow zone with C value ≥ 4^a^	0–3	4–9	10–12	≥13
Average C value^a^	0.00–2.50	2.51–3.57	3.58–4.58	≥4.59
Floristic Quality Index (FQI)^b^	0.00–13.60	13.61 −21.70	21.71 −27.20	≥27.21
Seasonally ponded				
Species richness of native perennials	0–19	20–31	32–41	≥42
Number of Genera of native perennials	0–14	15–24	25–32	≥33
Number of native grass and grass-like species	0–6	7–10	11 −17	≥18
Percentage of annual, biennial, and introduced species	≥41.1	30.8–41.0	21.1–30.7	0.0–21.0
Number of native perennial species in wet-meadow zone	0–8	9–16	17–24	≥25
Number of species with C value ≥ 5^a^	0–7	8–17	18–26	≥27
Number of species in the wet-meadow zone with C value ≥ 4^a^	0–4	5–9	10–16	≥17
Average C value^a^	0.00–2.60	2.61–3.12	3.13–3.52	≥3.53
Floristic Quality Index (FQI)^b^	0.00–10.00	10.01 −16.11	16.12–22.99	≥23.00

a Coefficients of conservatism (C value) follow the Northern Great Plains Floristic Quality Assessment Panel (2001).

b FQI, average C-value multiplied by the square root of the total number of native plant species (DeKeyser et al., 2003).

**TABLE 2:** Score ranges for each Index of Plant Community Integrity condition category for temporarily ponded and seasonally ponded wetlands of the Prairie Pothole Region of North America (see Hargiss et al., 2008).

	Score range	
Wetland condition	Temporarily ponded	Seasonally ponded
Very poor	--	0–19
Poor	0–33	20–39
Fair	34–66	40–59
Good	67–99	60–79
Very good	--	80–99

## Data Availability

The raw data supporting the conclusion of this article will be made available by the authors, without undue reservation.
